# Two Dominant Herbaceous Species Have Different Plastic Responses to N Addition in a Desert Steppe

**DOI:** 10.3389/fpls.2022.801427

**Published:** 2022-04-26

**Authors:** Aixia Guo, Xiaoan Zuo, Ya Hu, Ping Yue, Xiangyun Li, Peng Lv, Shenglong Zhao

**Affiliations:** ^1^Urat Desert-Grassland Research Station, Northwest Institute of Eco-Environment and Resources, Chinese Academy of Sciences, Lanzhou, China; ^2^University of Chinese Academy of Sciences, Beijing, China; ^3^Key Laboratory of Stress Physiology and Ecology in Cold and Arid Regions, Gansu Province, Lanzhou, China; ^4^Naiman Desertification Research Station, Northwest Institute of Eco-Environment and Resources, Chinese Academy of Sciences, Lanzhou, China; ^5^College of Resources and Environmental Engineering, Tianshui Normal University, Tianshui, China

**Keywords:** nitrogen addition, morphological traits, physiological traits, leaf anatomical structure, phenotypic plasticity, desert steppe

## Abstract

Nitrogen (N) deposition rates are increasing in the temperate steppe due to human activities. Understanding the plastic responses of plant dominant species to increased N deposition through the lens of multiple traits is crucial for species selection in the process of vegetation restoration. Here, we measured leaf morphological, physiological, and anatomical traits of two dominant species (*Stipa glareosa* and *Peganum harmala*) after 3-year N addition (0, 1, 3, and 6 g N m^−2^ year^−1^, designated N0, N1, N3, and N6, respectively) in desert steppe of Inner Mongolia. We separately calculated the phenotypic plasticity index (PI) of each trait under different N treatments and the mean phenotypic plasticity index (MPI) of per species. The results showed that N addition increased the leaf N content (LNC) in both species. N6 increased the contents of soluble protein and proline, and decreased the superoxide dismutase (SOD) and the peroxidase (POD) activities of *S. glareosa*, while increased POD and catalase (CAT) activities of *P. harmala*. N6 increased the palisade tissue thickness (PT), leaf thickness (LT), and palisade-spongy tissue ratio (PT/ST) and decreased the spongy tissue–leaf thickness ratio (ST/LT) of *S. glareosa*. Furthermore, we found higher physiological plasticity but lower morphological and anatomical plasticity in both species, with greater anatomical plasticity and MPI in *S. glareosa* than *P. harmala*. Overall, multi-traits comparison reveals that two dominant desert-steppe species differ in their plastic responses to N addition. The higher plasticity of *S. glareosa* provides some insight into why *S. glareosa* has a broad distribution in a desert steppe.

## Introduction

Atmospheric nitrogen (N) deposition has increased dramatically in recent decades due to human activities, such as fossil fuel combustion and chemical fertilizer application (Galloway et al., 2008; Zhang H. et al., 2020), and this trend is predicted to continue unabated, with the rate of N deposition doubling by 2050 (Fowler et al., 2013). N deposition events have occurred in temperate regions including northern China (Yue et al., 2018). Although N addition can promote plant photosynthetic capacity by enhancing the N content of leaves, excessive N deposition has been found to adversely affect plant traits and structure in terrestrial ecosystems, eventually triggering vegetation vulnerability to environmental stresses (Borghetti et al., 2017; Zhang et al., 2018; Zhang H. et al., 2021). Effects of atmospheric N deposition on ecosystem functioning are usually predicted based on shifts in the plant functional traits (Garnier et al., 2007; Hu et al., 2019), which are morphological, physiological, and phenological characteristics of plants (Pérez-Harguindeguy et al., 2016; Griffith et al., 2020). Since a series of functional traits that can represent the ecological niche space of species (McGill et al., 2006), they typically vary interspecifically and ought to be used for predicting how individual plant species respond to environmental change and model future changes in vegetation composition (Soudzilovskaia et al., 2013; Liancourt et al., 2015). Plants can respond to external environmental stress by adjusting their leaf morphological, physiological, and anatomical traits (Sherrard et al., 2009; Gratani et al., 2014; Guo et al., 2017).

The addition of N can cause changes in the leaf economic traits (Zheng et al., 2017). The study has shown that N addition increased leaf N content (LNC) of C_3_ and C_4_ grass species in a semi-arid grassland and further enhanced their photosynthetic capacity (Zhong et al., 2019). Recent work found that various N addition increased plant height, specific leaf area (SLA), leaf N concentration (LNC), and length, but decreased leaf dry matter content (LDMC) and carbon (C) to N ratio in the leaf of dominant species in a meadow steppe of Inner Mongolia, Northern China (Bai et al., 2020). Plant functional traits should be predictive for the vulnerability of a species in response to global change scenarios as their ample physiological bases (Liancourt et al., 2015; Pérez-Harguindeguy et al., 2016), such as those concerning photosynthesis, osmoregulation, and enzyme activities, can be used to explain the rapid adjustment ability of species and to understand better plant survival strategies in response to environmental stress (Singh et al., 2015; Boukili and Chazdon, 2017; Bachle and Nippert, 2018). Medium to high levels of N can impair osmolyte accumulation, photosynthesis, nitrate reductase activity, and reduced soluble protein, but low to medium levels of N enhanced proline content (Zhang Z. et al., 2021). An experimentally increased N deposition was found to enhance the leaf area, plant height, and chlorophyll content of both wheats and *Aegilops tauschii*, as well as their superoxide dismutase (SOD) activity and the reactive oxygen concentration (Wang and Chen, 2019). A previous study suggested an increase in leaf soluble sugar content and a decrease in starch concentration with N addition. Physiological traits may be directly influenced by species’ micro-anatomical features (Bachle and Nippert, 2018; Zheng et al., 2019). Leaf anatomy can determine the photosynthetic rate due to the complex structure and composition of the leaf organ (Evans et al., 2009). For example, the study suggested that the main limitation to optimizing photosynthesis is spongy and palisade tissue (Terashima et al., 2011). Meanwhile, changes in leaf thickness, mesophyll structure, the density, and size of stomata could affect photosynthesis and transpiration (Sun et al., 2014). Exploring the anatomical responses of plant leaves to N addition is needed now to gain a deeper understanding of how desert-steppe plants adapt to the changing environmental conditions (Cai et al., 2017). Plant traits are usually co-varied and are interrelated, so studying multiple traits can provide important insight into the adaptive processes of locally dominant plant species to climatic changes (Cadotte et al., 2015).

Phenotypic plasticity is the ability of plants to change their phenotype in response to environmental changes (Draghi and Whitlock, 2012; Lin et al., 2020; Rovelli et al., 2020). It determines the short-term ecological response of species to global changes; in some cases, it may directly buffer the adverse impact of these changes (Binks et al., 2016). Plant plastic responses to shifts in N deposition were triggered by the coordinated adjustment of morphological, physiological, and biochemical traits (Wang and Chen, 2019). Species’ leaf traits and their plasticity can indicate their ability to adjust to new environmental changes in the future (Gratani et al., 2014; Lin et al., 2020). Quantifying phenotypic plasticity index is necessary to compare the plasticity across traits and species (Valladares et al., 2000a,b, 2006; Sanchez-Gomez et al., 2006). The plasticity of physiological traits in *Halimium halimifolium* was higher than that of morphology and allocation traits, with the plasticity of most morphological traits being low (Zunzunegui et al., 2009; Ren et al., 2020). *Sesleria nitida* exhibited greater plasticity in physiology than in morphology or in anatomy (Valladares et al., 2000b; Gratani et al., 2014). The higher mean plasticity of species can reflect its adaptive ability to future global changes (Quan et al., 2015).

Desert steppe (i.e., highly arid grassland) is one of Inner Mongolia’s grassland ecosystems, accounting for 10.7% of its entire grasslands’ coverage (Jia et al., 2017) and forming the key transition area between grassland and desert (Zhang G. et al., 2020). Plants of desert steppe might be more vulnerable and responsive to climate change than are species in other grassland types (Vale and Brito, 2015). *Stipa glareosa* and *Peganum harmala*, two dominant species of the grass community in the desert steppe of Inner Mongolia, play critical roles in maintaining the function and structure of plant communities (Hu et al., 2020). However, how these two dominant desert-steppe species at the leaf level adjust and respond to locally increased N deposition remains unclear.

In this study, we sought to investigate how experimentally outdoors simulated N deposition levels may affect leaf-level morphological, physiological, and anatomical traits of *S. glareosa* and *P. harmala* in desert steppe of Inner Mongolia, as well as differential responses of these two dominant species in response to changing N levels. Based on the trait plasticity of the two species, another objective of this work was to select which of the two species is a better indicator of future global change in the study region.

## Materials and Methods

### Experimental Site and Design

The field experiment was conducted in desert steppe located at the Urat Desert-Grassland Research Station, the Chinese Academy of Science (41°25' N, 106°58′ E), lying at an elevation of 1720 m a.s.l. The site has a typical temperate continental climate with a mean annual temperature of 3.8°C. The annual precipitation averages 170 mm, of which about 80% falls during the growing season. The vegetation is dominated by *S. glareosa* (Gramineae, herbaceous perennials) and *P. harmala* (Zygophyllaceae, herbaceous perennials). The soil in this region is gravel brown calcic soil.

The N addition experiment was set up in May 2018, using a completely randomized block design with five replicates. There were 20 plots in total, each plot being 6 m × 6 m. N, in the form of urea, was evenly added to the plots at four N levels: 0 g N m^−2^ year^−1^ (control, N0), 1 N m^−2^ year^−1^ (N1), 3 g N m^−2^ year^−1^ (N3), and 6 g N m^−2^ year^−1^ (N6). These four N levels applied within a block—that is, spatially, each block had four plots, corresponding to the four N levels. Adjacent plots within a block were separated by a 1-m buffer zone. The annual application was divided into two equal doses and applied monthly from mid-June to mid-July. During each application, the urea was dissolved in purified water for even spraying across plots. The control plots received the same dose of water but without the urea in them. This region was not disturbed by grazing or weeding.

### Measurement of Morphological Traits

Two dominant species (*S. glareosa* and *P. harmala*) were selected for the present field study. Four key morphological traits of plants related to resources acquisition and use and strategy (Supplementary Table S1): SLA, LDMC, LNC, and leaf C content (LCC) were determined by the following methods (Cornelissen et al., 2003). From each plot, 15 healthy intact leaves on five individuals per species were randomly collected, immediately sandwiched in self-sealing bags containing moist filter paper, and kept in a car-powered refrigerator at 5°C for 12 h. Once in the laboratory, any moisture on the surface of leaves was then rapidly absorbed with absorbent paper. Leaf fresh weight was weighted on a Millionth electronic balance, and leaves were spread on a scanner to measure leaf areas (LAs) using Image J 1.8.0 software. Leaf dry weight was measured after owning all leaves at 65°C for 48 h and weighed. SLA was calculated as LA divided by leaf dry weight, and LDMC was calculated as leaf dry weight divided by fresh weight (Garnier et al., 2001). Leaves of each plant were collected dried at 65°C for 48 h (dry weight nearest to be 0.5 mg) to measure C and N contents in each plot. LCC and LNC were analyzed using an elemental analyzer (Costech ECS 4010, Italy).

### Measurement of Physiological Traits

The fully expanded, mature leaves (*n* = 10 per population) of two dominant species were randomly collected and stored at 4°C in a refrigerator to measure the physiological parameters (Supplementary Table S1). All absorbance values below are read by a UV–VIS spectrophotometer (SHIMADZU UV-1780).

### Chlorophyll Content

The measurement of chlorophyll content was performed according to the method described by Arnon (1949). Chlorophyll from the leaf samples (0.2 g fresh weight) was extracted using 95% (v/v) ethanol and was determined light absorption values of extracting solution at 665 nm and 649 nm. The chlorophyll a (Chl a), chlorophyll b (Chl b) contents (mg g^−1^), and chlorophyll a/b (Chl a/b) were calculated as:


$$\begin{array}{l} \mathrm{Chl}\;a=\left(13.95A665-6.88A649\right)\times V/W\\ \mathrm{Chl}\;b=\left(24.96A649-7.32A665\right)\times V/W\\ \mathrm{Chl}\;a/b=\mathrm{Chl}\;a/\mathrm{Chl}\;b \end{array}$$

In the formula, V is volume of extracting solution. W is leaf fresh weight.

### Starch, Soluble Sugar, and Soluble Protein Contents

Starch content was measured with a starch content assay kit (Solarbio, Beijing, China). A 0.1 g leaf samples was homogenated in an ice-cold mortar and extracted with the reagent. The mixture was boiled in a water bath at 80°C for 30 min, then centrifuged at 3,000 *g* for 5 min. The supernatants were removed and the precipitates were added into double distilled water, mixed, and centrifuged at 3,000 *g* for 10 min. The absorbance of supernatants was measured at 620 nm.

Soluble sugar content was determined with a soluble sugar content assay kit (Solarbio, Beijing, China). Leaf fresh samples (0.1–0.2 g) were weighted and homogenated in an ice-cold mortar with 1 ml distilled water. The mixture was boiled in a water bath at 100°C for 10 min, then cooled, and centrifuged at 8,000 *g* for 10 min. The supernatants were transferred to a 10 ml test tube, diluted it to 10 ml with distilled water, and were added into reagents and concentrated sulfuric acid. The absorbance was measured at 620 nm.

Soluble protein was determined using the biuret method of protein detection kit (Solarbio, Beijing, China). A 0.1 g leaf samples were homogenated in an ice-cold mortar with 1 ml distilled water, then centrifuged at 8,000 *g*, 4°C for 10 min. The supernatants were added into reagent and mixed. The absorbance was measured at 540 nm.

### Antioxidant Enzymes, Malondialdehyde, and Proline Contents

The assessment of SOD activity and peroxidase (POD) activity was carried out using corresponding test kits (Solarbio, Beijing, China). A 0.1 g leaf samples was homogenated with 1 ml extracting solution, then centrifuged at 8,000 *g*, 4°C for 10 min. The supernatants were added into reagents, mixed, and boiled in a water bath at 37°C for 30 min. The absorbance of SOD was measured at 560 nm. Similarly, the supernatants were added into reagents and mixed, and the absorbance of POD was measured at 470 nm, 30 s and 90 s, respectively. Catalase (CAT) activity was measured using CAT test kits (Keming, Suzhou, China). A 0.1 g leaf samples was homogenated with 1 ml extracting solution, then centrifuged at 8,000 *g*, 4°C for 10 min. The supernatants were added into reagent and mixed and the absorbance was measured at 405 nm.

Malondialdehyde (MDA) content was assayed following the method of MDA content test kit (Solarbio, Beijing, China). A 0.1 g leaf samples was homogenated with 1 ml extracting solution, then centrifuged at 8,000 *g*, 4°C for 10 min. The supernatants were added into reagents, mixed, boiled at 100°C for 90 min, then cooled, and centrifuged at 10,000 *g* for 10 min. The absorbance of supernatants was measured at 450 nm, 532 nm, and 600 nm.

Determination of proline was performed according to Bates et al. (1973). A 0.2 g leaf samples was homogenated and extracted with 80% ethanol. The homogenate was transferred to a 10 ml test tube, mixed, and boiled at 80°C for 20 min. A 2 ml extracting solution was added into 2 ml glacial acetic acid and ninhydrin reagent, boiled at 100°C for 15 min. The absorbance was measured at 520 nm.

### Relative Water Content

The relative water content (RWC) was measured according to Barrs and Weatherley (1962). The fresh leaves were quickly cut into small pieces and weighed for their fresh weight (Wf: to be nearest 1 g), and then immerse in distilled water for 6–8 h. Moisture on the surface of leaves was absorbed with absorbent paper. The leaves were weighed and then immersed again in distilled water for 1 h. The above steps were repeated until a constant weight of leaves was reached, that is, a saturated fresh weight (Wt). Leaves were quenched in an oven at 105°C for 15 min and then dried at 80°C to a constant weight to obtain their dry weight (Wd). The relative water content (RWC) of leaves was then calculated using the following equation:


RWC=(Wf−Wd)/(Wt−Wd)×100

### Measurement of Leaf Anatomical Traits

Leaf anatomical structure was evaluated *via* paraffin sectioning of tissues and light microscopy observations/methods (Spannl et al., 2016). We selected fully expanded sun leaves from five randomly selected plants of each species. These leaves were washed with distilled water and cut into squares of 5–10 mm, and quickly fixed for 48 h in a formalin-acetic acid-alcohol solution (FAA: 90 ml of 70% alcohol, 5 ml of glacial acetic acid, and 5 ml of 40% formalin). Leaves were progressively dehydrated with ethanol series (50%, 70%, 85%, 95%, 100%, and 100% for 2 h per percentage), cleared with xylene, and embedded in warm paraffin (52°C–54°C and 56°C–58°C). Leaf transverse sections were cut into thin sections (8–12 μm thickness) using a rotary microtome (KD2260, China) and attached to the glass slide with gelatin and placed in an oven at 37°C for 48 h. The sections were de-waxed with xylene and then gradually passed through a gradient of alcohol (100%, 100%, 95%, 85%, 70%, and 50% for 1 h per percentage) until distilled water. The samples were stained with safranin-fast green and their anatomical structures were observed under light microscopy using an image analysis system (Axiovert A1, Zeiss). We calculated six trait variables in Digimizer software: palisade tissue thickness (PT), spongy tissue thickness (ST), leaf thickness (LT), palisade-spongy tissue ratio (PT/ST), palisade tissue–leaf thickness ratio (PT/LT), and spongy tissue–leaf thickness ratio (ST/LT) (Supplementary Table S1).

### Phenotypic Plasticity

Trait plasticity for each trait and plant species was expressed by the phenotypic plasticity index (PI), calculated as follows (Valladares et al., 2000b):


$$\mathrm{PI}=\frac{\text{Max}\left(Ni,N0\right)-\text{Min}\left(Ni,N0\right)}{\text{Max}\left(Ni,N0\right)}$$

where Ni and N0 are the mean values of a trait for one plant species in a given N addition treatment and the control treatment, respectively. Max (Ni and N0) represents the largest values of Ni and N0, while Min (Ni and N0) denotes the smallest values of Ni and N0. PI ranges from zero to one. Zero and one, respectively, denote no plasticity and maximum plasticity. We first calculated the PI of each trait for different N treatments. We then calculated the corresponding plasticity index of the morphological (PI_m_), physiological (PI_p_), and anatomical leaf traits (PI_a_) for different N treatments and also for all treatments. Finally, the mean phenotypic plasticity index (MPI) of each species was calculated by averaging the PI_m_, PI_p_, and PI_a_ (Gratani et al., 2014).

### Measurement of Soil Properties

Soil samples (0–20 cm) were collected and repeated three times in each plot to measure soil properties. One part of the samples was oven-dried at 105°C for 12 h to measure soil water content (WC). After air drying, other samples were sifted through a 0.5-mm sieve and then determine the chemical properties. Soil pH and electrical conductivity (EC) were measured at a soil:water ratio of 1:5 (w/v) using a pH and an EC meter (SX823; LABSEN Scientific Instrument, Shanghai, China). The soil total C and N were determined by an elemental analyzer (Costech ECS4010, Italy). Bulk density (BD) was measured following the method (Li et al., 2012). Soil texture (coarse sand, medium sand, fine sand, fine sand, clay, and silt) was measured following the method (Lv et al., 2021).

### Statistical Analysis

Two-way ANOVA was used to analyze the effects of species, N addition, and their interactions on leaf traits, PI, as well as the effects of species, trait types, and their interactions on MPI. One-way ANOVA was used to analyze separate effects of species and N addition on leaf traits, PI, as well as the effects of species and trait types on MPI, and multiple comparisons were performed by least-significant difference (LSD; *p* < 0.05) test. Redundancy analysis (RDA) was performed to assess the effects of soil properties on leaf traits. All statistical analyses were performed using SPSS 22.0 for Windows (SPSS Inc., Chicago, Illinois, United States). All data in the Figures are presented as the mean ± SE. Figures were produced in Origin 9.1 software (Origin Lab, Hampton, MA, United States). The RDA was performed in Canoco 5.0.

## Results

### Effects of Increased N Addition on Plant Morphological Traits

**Figure 1:**
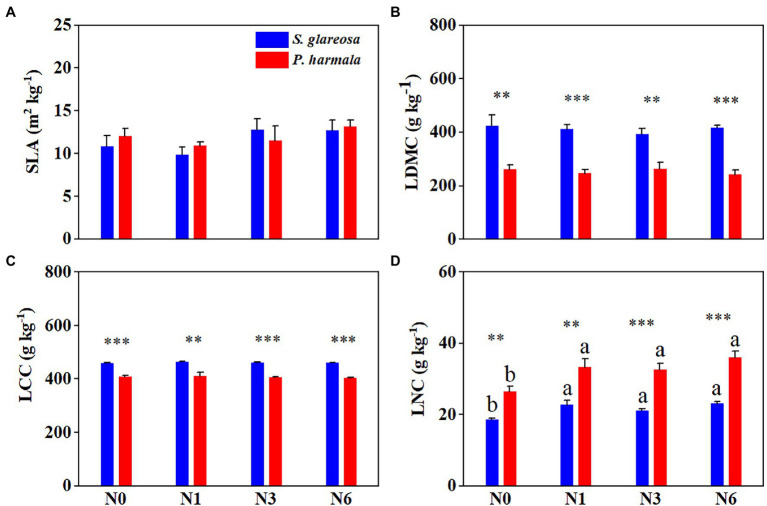
Response of leaf morphological traits in *Stipa glareosa* and *Peganum harmala* to the different N treatments. **(A)** Specific leaf area (SLA), **(B)** leaf dry matter content (LDMC), **(C)** leaf carbon content (LCC), and **(D)** leaf nitrogen content (LNC). Values are presented as the means ± SEs (*n* = 5). Different lower-case letters represent significant differences among the treatments for each species (*p* < 0.05). Asterisks represents significant differences between *S. glareosa* and *P. harmala* (^**^p < 0.01, and ^***^p < 0.001).

Species significantly affected LDMC, LCC, and LNC. N addition had a significant effect on LNC of *S. glareosa* and *P. harmala*, and the interaction between species and N addition did not have statistical effects on morphological traits (Supplementary Table S2). N addition statistically increased LNC of *S. glareosa* and *P. harmala* (Figure 1D). Under different N treatments, significant differences in traits were evident between the two species, except for SLA (Figures 1A–D). The LDMC and LCC of *S. glareosa* were higher than that of *P. harmala*, but the LNC was lower in *S. glareosa* than *P. harmala*.

### Effects of Increased N Addition on Plant Physiological Traits

**Figure 2:**
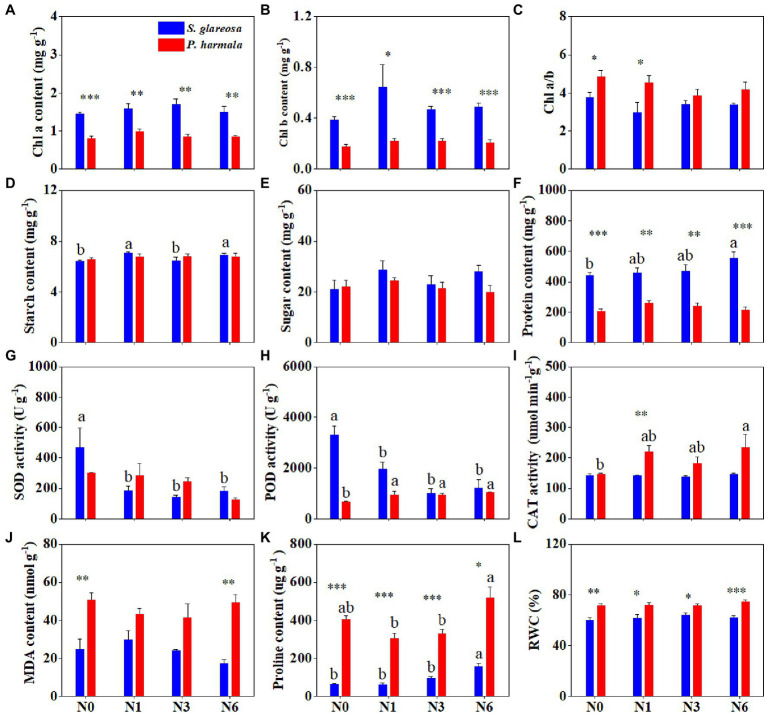
Response of leaf physiological traits in *S. glareosa* and *P. harmala* to different N treatments. **(A)** Chlorophyll a (Chl a) content, **(B)** chlorophyll b (Chl b) content, **(C)** chlorophyll a-to-b ratio (Chl a/b), **(D)** starch content, **(E)** soluble sugar content, **(F)** soluble protein content, **(G)** superoxide dismutase (SOD) activity, **(H)** peroxidase (POD) activity, **(I)** catalase (CAT) activity, **(J)** malondialdehyde (MDA) content, **(K)** proline content, and **(L)** relative water content (RWC). Different lower-case letters represent significant differences among the treatments for each species (*p* < 0.05). Asterisks represent significant differences between *S. glareosa* and *P. harmala* (^*^p < 0.05; ^**^p < 0.01; and ^***^p < 0.001).

Species had a significant effect on physiological traits, except starch, soluble sugar, and SOD. N addition significantly affected SOD, POD, and proline of *S. glareosa* and *P. harmala*, and their interaction significantly affected POD and proline content (Supplementary Table S2). We found that different N addition rates had negligible effects on Chl a, Chl b, and Chl a/b of both species but Chl a and Chl b of *S. glareosa* were significantly higher than that of *P. harmala* (Figures 2A–C). N addition had no effects on the contents of starch, soluble sugar, and protein of *P. harmala* and soluble sugar of *S. glareosa* (Figures 2D–F). N6 significantly increased the contents of starch and soluble protein of *S. glareosa*. Significant differences of soluble protein content between two species were detected, being higher for *S. glareosa* than *P. harmala* (Figure 2F). N addition decreased SOD and POD activities in *S. glareosa* (Figures 2G,H). N6 significantly increased the POD and CAT activities of *P. harmala*, and the proline content of *S. glareosa* (Figures 2H,I,K). N addition has no discernible effects on the MDA and RWC of either *S. glareosa* or *P. harmala* (Figures 2J,L). Significant differences of proline content and RWC were found between two species and these two traits of *P. harmala* were higher than that of *S. glareosa* (Figures 2K,L).

### Effects of Increased N Addition on Plant Anatomical Traits

**Figure 3:**
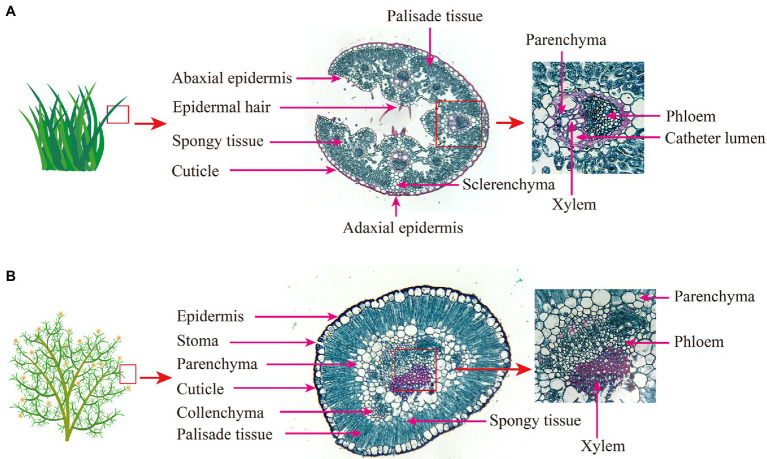
Light microscopy images of the leaf transverse profile for **(A)** *S. glareosa* and **(B)** *P. harmala* under control (N0) treatment. The images were taken under a magnification of 10× (left) and 40× (right).

**Figure 4:**
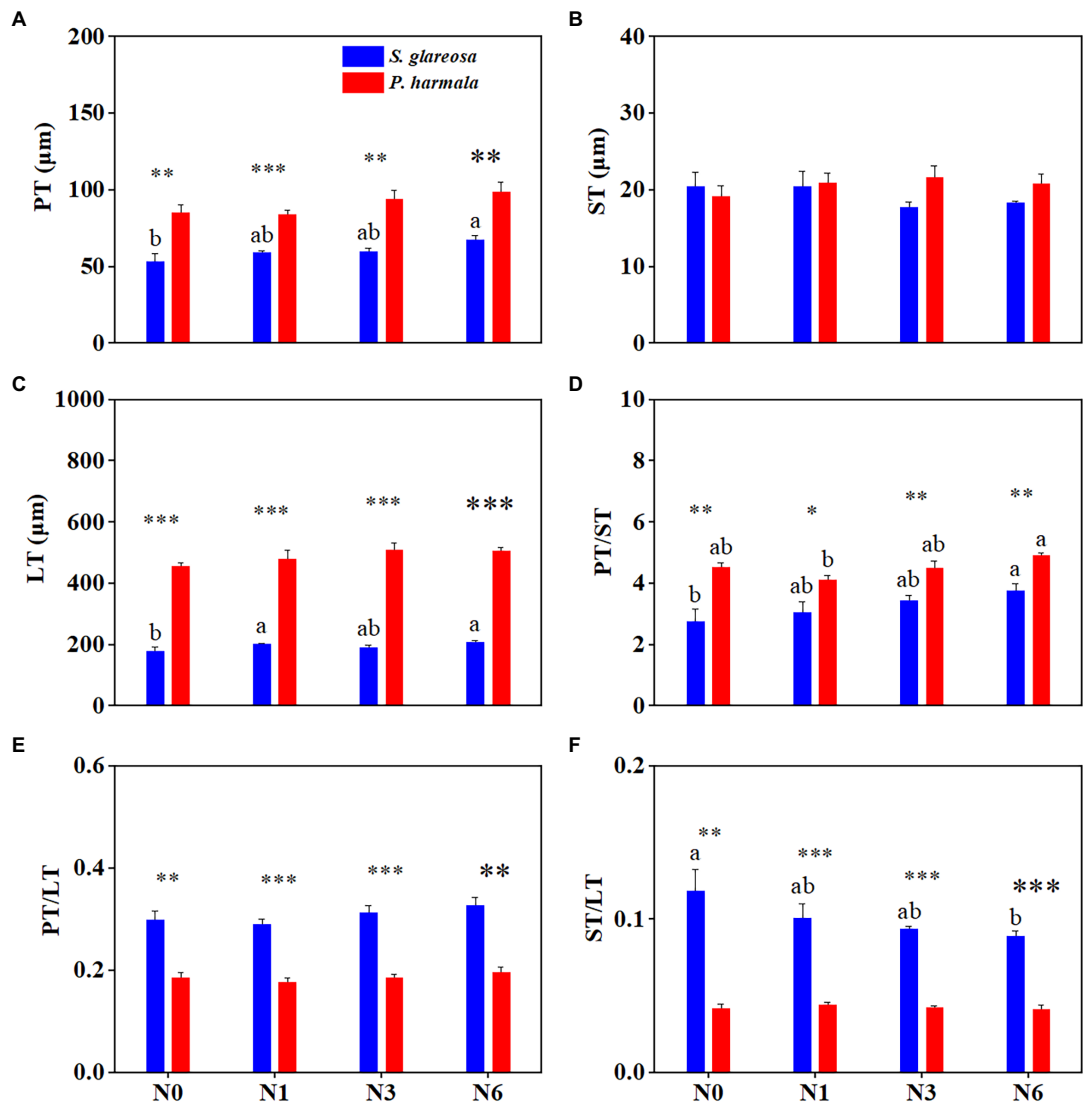
Response of leaf anatomical traits in *S. glareosa* and *P. harmala* to different N treatments. **(A)** Palisade tissue thickness (PT), **(B)** spongy tissue thickness (ST), **(C)** leaf thickness (LT), **(D)** palisade–spongy tissue ratio (PT/ST), **(E)** palisade tissue–leaf thickness ratio (PT/LT), and **(F)** spongy tissue–leaf thickness ratio (ST/LT). Different lower-case letters represent significant differences among the treatments for each species (*p* < 0.05). Asterisks represent significant differences between *S. glareosa* and *P. harmala* (^*^p < 0.05; ^**^p < 0.01; and ^***^p < 0.001).

Species had a significant effect on anatomical traits, except ST. N addition significantly affected PT and PT/ST, and their interaction had no significant effects on anatomical traits (Supplementary Table S2). Differences in the anatomical structure of *S. glareosa* (Figure 3A) and *P. harmala* (Figure 3B) were found. Varying the level of N addition had statistically no effects upon the anatomical traits of *P. harmala* (Figures 4A–F). When compared with N0, applying N6 significantly increased the PT, LT, and PT/ST and decreased the ST/LT of *S. glareosa* (Figures 4A,C,D,F). Apart from ST, other traits values differ significantly between *S. glareosa* and *P. harmala*, with PT, LT, and PT/ST significantly higher in *P. harmala*.

### Effects of Increased N Addition on Trait Phenotypic Plasticity

**Figure 5:**
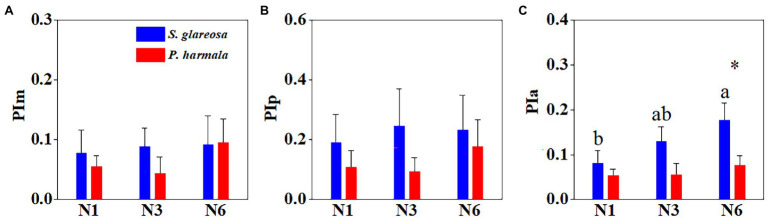
Mean phenotypic plasticity indexes of all traits of *S. glareosa* and *P. harmala* under different N treatments divided by trait types. **(A)** Phenotypic plasticity indexes of morphological traits; **(B)** phenotypic plasticity indexes of physiological traits; and **(C)** phenotypic plasticity indexes of anatomical traits. Different lower-case letters represent significant differences within each treatment for each species (*p* < 0.05). Asterisk represents significant differences between *S. glareosa* and *P. harmala* (*p* < 0.05).

**Figure 6:**
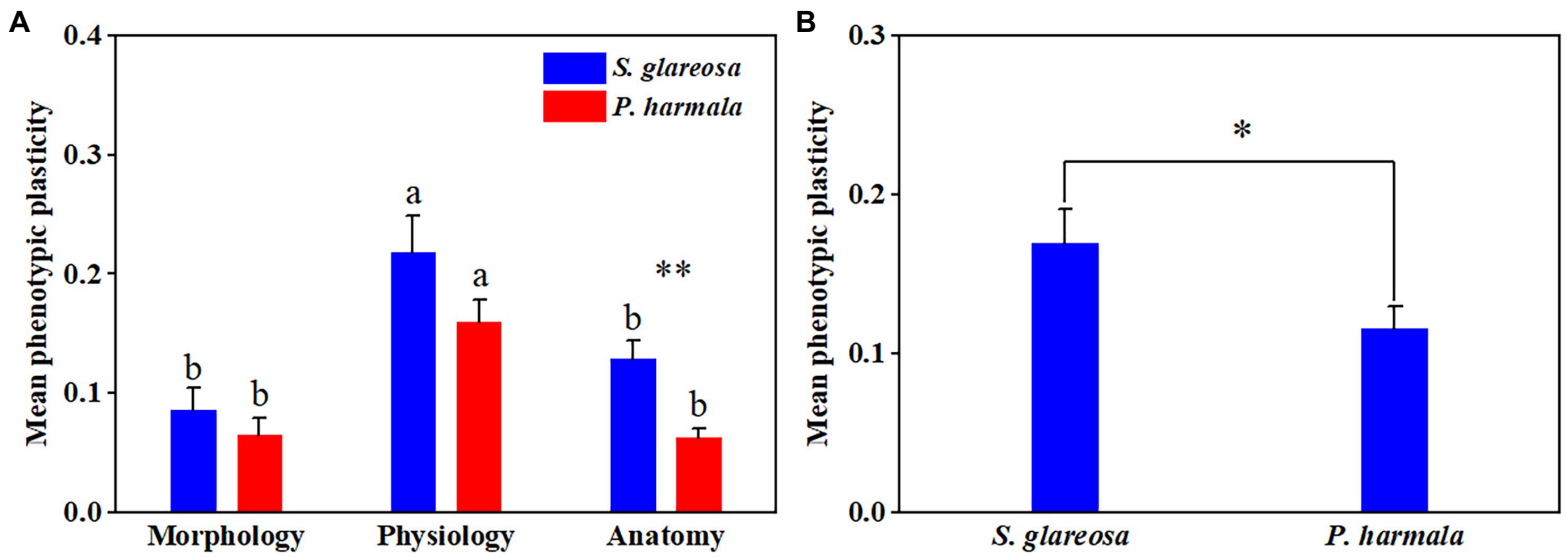
Mean phenotypic plasticity of *S. glareosa* and *P. harmala*. **(A)** Mean phenotypic plasticity of all treatments of *S. glareosa* and *P. harmala* divided by trait types. **(B)** Total mean phenotypic plasticity of *S. glareosa* and *P. harmala*. Different lower-case letters represent significant differences within each traits types for each species (*p* < 0.05). Asterisk represents significant differences between *S. glareosa* and *P. harmala* (^*^p < 0.05 and ^**^p < 0.01).

PI of each trait and species were calculated under the different N addition treatments (Supplementary Table S3). Averaged plasticity indexes of PIm, PIp, and PIa, under the different treatments were then compared (Figure 5). Species and N addition significantly affected PIa, and their interaction had no significant effects on PI (Supplementary Table S4). The three PIs were negligibly affected by the different N treatments in *P. harmala* (Figures 5A–C). The PIa of *S. glareosa* under the N6 treatment surpassed that under the other N treatments, and this index differed between the two species under the N6 treatment (Figure 5C). Then, MPI of morphological, physiological, and anatomical of all treatments was then analyzed (Figure 6A). Trait types significantly affected MPI of *S. glareosa* and *P. harmala*, and species and the interaction had no significant effects on MPI (Supplementary Table S4). The MPI of physiology of *S. glareosa* and *P. harmala* was each significantly higher than their respective MPI of morphology and anatomy; the last differed significantly between the two species. Finally, the MPI of each species was obtained (Figure 6B); it was significantly higher for *S. glareosa* than *P. harmala*.

### Correlations Between Leaf Traits and Soil Properties

**Figure 7:**
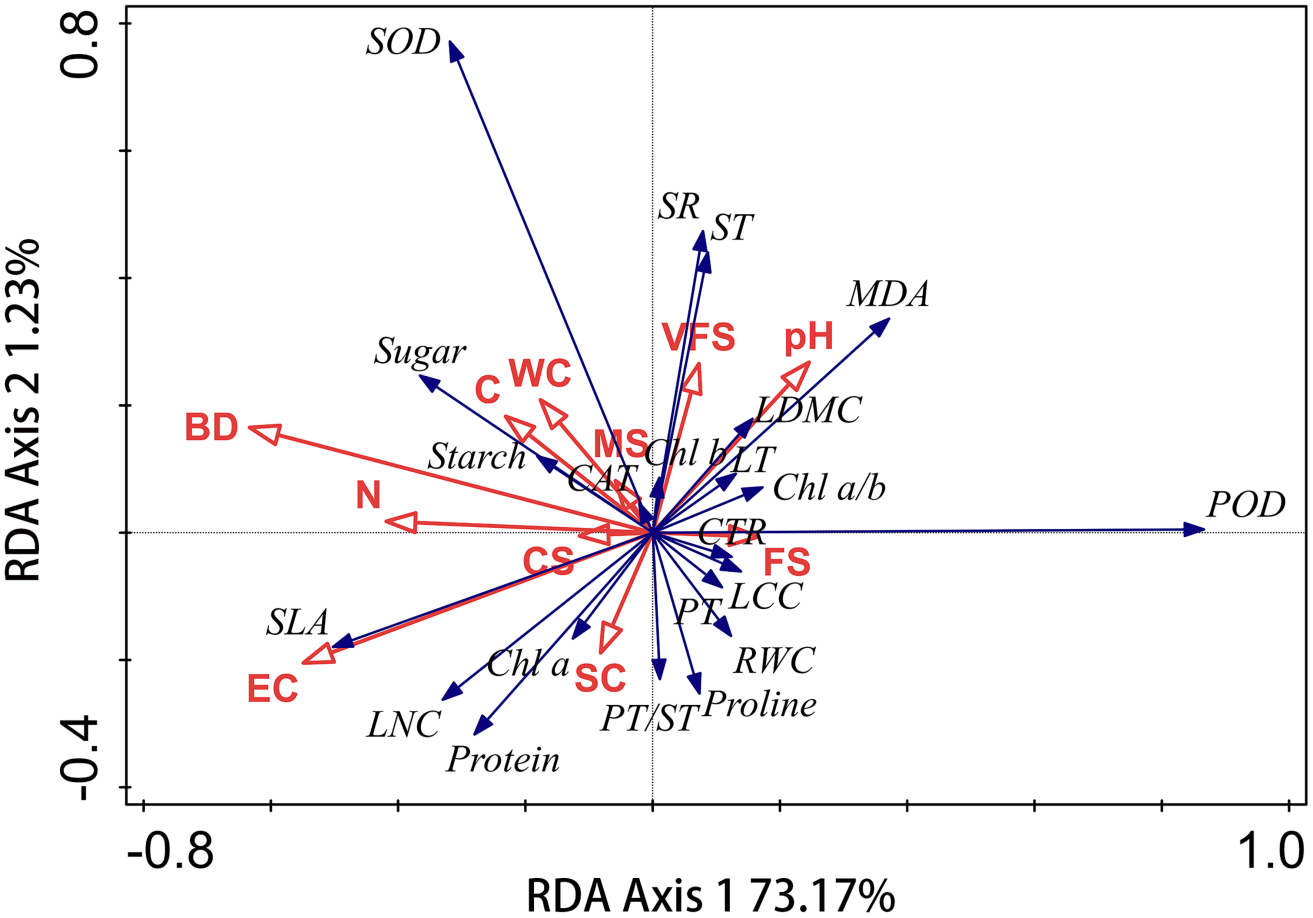
Redundancy analysis (RDA) identifying the effects of soil properties on leaf traits of *S. glareosa*. WC, water content; EC, electrical conductivity; BD, bulk density; CS, coarse sand; MS, medium sand; FS, fine sand; VFS, very fine sand; and SC, silt and clay.

Changes in leaf traits of plant species may be affected by soil environmental factors. The correlations of leaf traits of *S. glareosa* and *P. harmala* and vis-à-vis soil properties were analyzed *via* RDA. In each RDA, leaf traits were used as the response variables and soil properties as the explanatory variables. Leaf traits of *P. harmala* did not affect by soil factors (Supplementary Figure S1; Supplementary Table S5). The RDA’s first and second axes explained 73.17% and 1.23% of the total variation in the leaf traits of *S. glareosa* (Figure 7). BD and EC significantly are the main factors that influenced leaf traits (Table 1).

**Table 1:** Contribution of soil properties to variation of leaf traits of *S. glareosa*.

Soil variables	Explains (%)	Contribution (%)	Pseudo-F	*p*
Bulk density (BD)	29.4	39.3	7.5	0.026
Electrical conductivity (EC)	15.0	20.1	4.6	0.046
Coarse sand (CS)	9.4	12.5	4.3	0.076
pH	8.8	11.7	3.0	0.094
N	7.1	9.5	2.7	0.116
C	1.6	2.1	0.7	0.420
Very fine sand (VFS)	1.3	1.7	0.5	0.522
Water content (WC)	1.0	1.4	0.4	0.540
Silt and clay (SC)	0.6	0.8	0.2	0.706
Fine sand (FS)	0.3	0.4	0.1	0.784
Medium sand (MS)	0.3	0.5	0.1	0.808

## Discussion

### Responses of Morphological Traits to N Addition

Morphological traits are a set of leaf traits connected to resource acquisition (Firn et al., 2019). These traits are thought to represent the stress resistance and competitiveness of the plant species. The SLA, LDMC, LCC, and LNC jointly convey resource exploitation and conservation trade-offs (Pierce et al., 2013). In the study, the LNC of both species showed susceptibility in response to different N addition levels. This result is consistent with previous findings wherein long-term N addition enhanced remarkably the leaf N concentration in the plant dominant species (Broadbent et al., 2020; Khan et al., 2020; Sun et al., 2022). Previous studies suggest that most species increased in SLA and declined in LDMC with the addition of N (Tatarko and Knops, 2018; Sun et al., 2022); however, the SLA, LDMC, and LCC of the two species did not respond to the N addition, and the reason for inconsistency with previous findings may be due to species-specific. The traits of the two species were differentially responsive to N addition, LDMC and LCC of *S. glareosa* were consistently higher than those of *P. harmala*, and LNC of the former was lower than the latter. The different responses of the two species reflect plant adaptation to habitats (Sun et al., 2022).

### Responses of Physiological Traits to N Addition

As the basis of the production and utilization of photosynthate, leaf physiological characteristics are closely related to internal chemical characteristics (Broadbent et al., 2020). Exploring how nutrients shape leaf physiological traits is one of the primary questions in ecology (Maire et al., 2015). Chlorophyll content reflects a certain extent the photosynthesis of plants (Zhang T. et al., 2016). In our study, the Chl a, Chl b, and Chl a/b of either species were not significantly affected by different N addition levels, which is in agreement with the earlier findings that Chl content did not respond to N addition (Arroniz-Crespo et al., 2008; Zhang et al., 2017). However, it has also been shown that N addition induced a significant increase in the chlorophyll content (Yao et al., 2016; Zhang Y. et al., 2016; Shen et al., 2020), suggesting that the response of chlorophyll to N addition depends on the species considered. The soluble protein content of *S. glareosa* was enhanced by N6 treatment, probably because N is a central component of proteins, can be directly absorbed by plant species and readily assimilated into protein for enhancing plant growth (Zhang Z. et al., 2021). Besides, the soluble protein content was higher in *S. glareosa* than *P. harmala*, indicating the stronger N uptake of the former. N6 also increased the starch content of *S. glareosa*, indicating that it stores photosynthetic products mainly in the form of starch for plant survival and growth under N6 conditions (Du et al., 2020).

The levels of reactive oxygen species (ROS) usually increased due to stress-induced disturbances in plant metabolism (Aguiar-Silva et al., 2016). Cell membrane lipid peroxidation can produce malondialdehyde and also reflect the accumulation of ROS (Zhou et al., 2018). Antioxidant enzyme activities are an important defense mechanism that influences stress tolerance in plants (Naudts et al., 2014). Abiotic stresses caused by certain excess nutrients addition can lead to increases in antioxidant enzyme activities (SOD, POD, and CAT; Medici et al., 2004). It has been found that all three enzyme activities were sensitive to N addition (Neves et al., 2009). Zhang et al. (2017) suggested low and medium N deposition significantly reduced POD activity but not SOD and CAT. Our results showed that SOD and POD in *S. glareosa* decreased in response to N addition, while POD and CAT in *P. harmala* increased in response to N6. These results suggest defense mechanisms against oxygen toxicity under N addition differ among species, and N deposition may enhance ROS scavenging capacity in *P. harmala*, and thus alleviate exterior stress on plant plasma membrane damage (Shen et al., 2020). Proline is an essential osmotic regulator that protects plants from oxidative stress. In general, the content of proline increases with increasing conditions of adversity faced by plants (Foyer and Noctor, 2005). Increased proline in *S. glareosa* likely protects it from damage caused by N6 addition. In addition, N addition did not cause a change in RWC of both species, suggesting that RWC may not be generally a valuable indicator of N addition impacts in desert-steppe species. In summary, *S. glareosa* adapted to the increased N addition by increasing osmolytes, such as soluble protein and proline contents, while *P. harmala* adapted to high N by increasing POD and CAT activities to eliminate free radicals in the body. These results reflect the instantaneous regulatory effects of physiological traits on *S. glareosa* and *P. harmala*, and differential physiological responses to increased N addition between the two species.

### Responses of Anatomical Traits to N Addition

Changes in anatomical structure reflect plant evolutionary adaptation to long-term environmental stresses (Guo et al., 2017). In the present study, N6 induced greater PT, LT, PT/ST, and diminished ST/LT of *S. glareosa*, suggesting that N6 enhanced the structural traits associated with photosynthesis and reduced looseness of leaves, which is similar to the results of the previous study that the anatomical traits of *Arabidopsis thaliana* were enhanced by N addition, satisfying the plant growth and nutrient requirements (Cai et al., 2017). N addition did not alter the anatomical traits of *P. harmala*. Hence, different co-occurring dominant species can exhibit differential response patterns to N addition in terms of structural traits. *S. glareosa* is sensitive enough to respond to the N6 addition, whereas *P. harmala* adopts a conservative strategy and does not respond to varying degrees of N addition.

### Responses of Phenotypic Plasticity to N Addition

Phenotypic plasticity can be indicative of the adaptive capability of a plant species to future global change (Nicotra et al., 2010), in that it allows a species to have a wider ecological range and endoes it with better stress tolerance. In this study, the highest anatomical plasticity of *S. glareosa* and a marked difference in plasticity among species was found for anatomical traits under the N6 treatment, suggesting that N6 is conducive to the formation of structural organization of plant species. In addition, the results of this study showcase higher plasticity for physiological traits than for morphological and anatomical ones, which agrees with the previous study that physiological plasticity plays a primary role (Gratani et al., 2014; Quan et al., 2015; Ren et al., 2020). The greater physiological plasticity ensured an instantaneous adjustment of plant species to changes in environmental factors intensity (Zunzunegui et al., 2009). Conversely, plants experiencing stress tend to have conservative leaf morphological patterns to avoid the structural changes that are too great to be sustained (Valladares et al., 2000b). *S. glareosa* exhibited greater mean phenotypic plasticity than *P. harmala*, indicating that *S. glareosa* can withstand a high environmental variation. It has also been shown that species with high plasticity have the ability to change their functional traits in response to environmental changes in their habitats (Zunzunegui et al., 2009).

### Relationships Between Leaf Traits and Soil Properties

The two dominant species are native to the desert steppe of Inner Mongolia and have adapted to long-term changes in their external environment. Given the greater phenotypic plasticity of *S. glareosa* than *P. harmala*, *S. glareosa* is more susceptible to other environmental variables such as soil factors. Variability in *S. glareosa* leaf traits is correlated with soil bulk density and electrical conductivity. This is consistent with the findings of a previous study (Joshi and Garkoti, 2021). Variation of plant traits can be attributed to adaptations to the environments, such as soil properties and climate change (Oliveira et al., 2019). However, the leaf traits of *P. harmala* depend little (i.e., are largely invariant) on soil nutrients under conditions of poor soil status and short-term environmental changes. The leaf traits of *P. harmala* may have a stronger relationship with precipitation during the growing season.

## Conclusion

This work demonstrates the differential plastic responses of two dominant desert-steppe species to N addition by studying multiple leaf traits. Physiological traits were more plastic and contributed to a greater extent to the acclimation ability of two species. *S. glareosa* was more responsive to increased N addition and had stronger plasticity than does *P. harmala*, which further explains *S. glareosa* has a wider ecological niche in desert steppe and thus can be selected as a predictor of grass species response to future climate change. Moreover, we inferred that the phenotypic plasticity may be higher in gramineae species than in non-gramineae species. However, this study involved only two dominant species, and we would like to experiment to strengthen this conclusion in the future by comparing the plasticity across multiple species.

## Data Availability Statement

The datasets presented in the study are included in the article/Supplementary Material; further inquiries can be directed to the corresponding author.

## Author Contributions

AG: conceptualization, investigation, formal analysis, writing—original draft, and writing—review and editing. XZ: conceptualization, writing—review and editing, and supervision. YH and XL: investigation and formal analysis. PY: investigation and writing—review and editing. PL and SZ: formal analysis and writing—review and editing. All authors contributed to the article and approved the submitted version.

## Funding

This study was financially supported by the Second Tibetan Plateau Scientific Expedition and Research program (2019QZKK0305) and National Natural Science Foundation of China (42071140).

## Conflict of Interest

The authors declare that the research was conducted in the absence of any commercial or financial relationships that could be construed as a potential conflict of interest.

## Publisher’s Note

All claims expressed in this article are solely those of the authors and do not necessarily represent those of their affiliated organizations, or those of the publisher, the editors and the reviewers. Any product that may be evaluated in this article, or claim that may be made by its manufacturer, is not guaranteed or endorsed by the publisher.
