# Supplementary material for: Two Dominant Herbaceous Species Have Different Plastic Responses to N Addition in a Desert Steppe
Source: Front Plant Sci. 2022 Apr 26;13:801427. doi: 10.3389/fpls.2022.801427 (PMC9087737; doi:10.3389/fpls.2022.801427)
Supplement: Supplementary file 1 [file Data_Sheet_1.docx]

Supplementary Material

# Supplementary Figures and Tables

## Supplementary Tables

**Table S1** Description of morphological, physiological and anatomical traits.

| Traits | Description |
| --- | --- |
| SLA | Related to plant growth and photosynthetic rates, |
| LDMC | Related to leaf resistance to physical stress and is also correlated with growth and photosynthetic rates |
| LCC | Primary productivity of plants |
| LNC | Absorption characteristics of soil nutrients; water use efficiency; photosynthetic rate of leaves |
| Chl a | Absorption of light energy; conversion of light energy |
| Chl b | Absorption of light energy |
| Chl a/b | Reflecting how much light energy is used by plants |
| Starch | Providing energy for the growth and development of plants and is a form of energy storage |
| Soluble sugar | A metabolic intermediate; regulating the growth and development of plants |
| Soluble protein | Important osmoregulation substances and nutrients, as one of the indicators for screening resistance |
| SOD | Scavenging of reactive oxygen species |
| POD | Scavenging of reactive oxygen species |
| CAT | One of the key enzymes of the biological defense system |
| MDA | Membrane lipid peroxidation production |
| Proline | Response to environmental stress; maintenance of homeostasis in cells |
| RWC | Effective moisture content in plants |
| PT | Plant use efficiency of water and light energy |
| ST | Reflecting the adaptation of plants to the environment |
| LT | Physical support capacity and water storage ability; light interception |
| PT/ST | Reflecting the resistance of plants to the environment |
| PT/LT | Tightness of leaf palisade tissue |
| ST/LT | Looseness of leaf spongy tissue |

**Table S2** The *F* values of two-way ANOVA for effects of species, N addition and their interactions on leaf morphological, physiological, and anatomical traits.

| Variables | Species | N | Species×N |
| --- | --- | --- | --- |
| SLA | 0.174 | 1.852 | 0.510 |
| LDMC | 97.271*** | 0.199 | 0.341 |
| LCC | 191.282*** | 0.305 | 0.177 |
| LNC | 110.455*** | 8.578*** | 1.064 |
| Chl a | 83.079*** | 0.980 | 0.614 |
| Chl b | 39.946*** | 1.924 | 1.031 |
| Chl a/b | 15.679*** | 1.547 | 0.855 |
| Starch | 0.015 | 1.970 | 1.250 |
| Soluble sugar | 2.566 | 1.284 | 0.928 |
| Soluble protein | 143.142*** | 1.474 | 2.171 |
| SOD | 0.018 | 4.571* | 2.024 |
| POD | 32.150*** | 6.263** | 11.166*** |
| CAT | 15.882*** | 2.487 | 2.134 |
| MDA | 35.185*** | 0.419 | 1.238 |
| Proline | 260.237*** | 11.272*** | 3.165* |
| RWC | 62.208*** | 0.727 | 0.744 |
| PT | 88.319*** | 3.455* | 0.381 |
| ST | 1.611 | 0.242 | 1.193 |
| LT | 624.872*** | 1.995 | 0.714 |
| PT/ST | 46.131*** | 3.291* | 0.867 |
| PT/LT | 172.614*** | 1.491 | 0.287 |
| ST/LT | 145.795*** | 1.772 | 1.729 |

Note: *, *p*＜0.05; **, *p*＜0.01; ***, *p* < 0.001

**Table S3** Phenotypic plasticity indexes of morphological, physiological, and anatomical traits of *S. glareosa* and *P. harmala* under different N treatments.

| Variables | *S. glareosa* | | | *P. harmala* | | |
| --- | --- | --- | --- | --- | --- | --- |
|  | N1 | N3 | N6 | N1 | N3 | N6 |
| Morphology | | | | | | |
| SLA | 0.086 | 0.154 | 0.149 | 0.091 | 0.041 | 0.085 |
| LDMC | 0.030 | 0.072 | 0.018 | 0.059 | 0.002 | 0.076 |
| LNC | 0.185 | 0.121 | 0.197 | 0.067 | 0.123 | 0.205 |
| LCC | 0.010 | 0.006 | 0.004 | 0.004 | 0.007 | 0.014 |
| Physiology | | | | | | |
| Chl a | 0.081 | 0.148 | 0.029 | 0.173 | 0.043 | 0.055 |
| Chl b | 0.401 | 0.173 | 0.208 | 0.219 | 0.224 | 0.170 |
| Chl a/b | 0.216 | 0.104 | 0.113 | 0.056 | 0.198 | 0.135 |
| Starch | 0.091 | 0.009 | 0.074 | 0.027 | 0.037 | 0.027 |
| Sugar | 0.269 | 0.077 | 0.246 | 0.095 | 0.042 | 0.101 |
| Protein | 0.031 | 0.064 | 0.203 | 0.211 | 0.156 | 0.050 |
| SOD | 0.598 | 0.702 | 0.611 | 0.045 | 0.176 | 0.585 |
| POD | 0.407 | 0.690 | 0.634 | 0.299 | 0.305 | 0.360 |
| CAT | 0.008 | 0.034 | 0.027 | 0.337 | 0.206 | 0.382 |
| MDA | 0.163 | 0.026 | 0.307 | 0.149 | 0.181 | 0.021 |
| Proline | 0.034 | 0.333 | 0.588 | 0.239 | 0.183 | 0.225 |
| RWC | 0.029 | 0.069 | 0.038 | 0.010 | 0.003 | 0.039 |
| Anatomy | | | | | | |
| PT | 0.095 | 0.109 | 0.211 | 0.012 | 0.095 | 0.141 |
| ST | 0.001 | 0.134 | 0.105 | 0.083 | 0.114 | 0.080 |
| LT | 0.121 | 0.067 | 0.137 | 0.044 | 0.099 | 0.098 |
| PT/ST | 0.096 | 0.202 | 0.270 | 0.091 | 0.006 | 0.075 |
| CTR | 0.025 | 0.048 | 0.088 | 0.043 | 0.004 | 0.051 |
| SR | 0.150 | 0.212 | 0.248 | 0.045 | 0.015 | 0.018 |

**Table S4** The *F* values of two-way ANOVA for effects of species, N addition/trait types and their interactions on trait phenotypic plasticity (PI) /mean phenotypic plasticity (MPI).

| Variables | Species | N | Species×N |
| --- | --- | --- | --- |
| PIm | 0.554 | 0.399 | 0.224 |
| PIp | 1.763 | 0.449 | 0.066 |
| PIa | 12.710** | 3.385* | 1.222 |
| Variables | Species | Trait types | Species×Trait types |
| MPI | 1.247 | 5.880** | 0.259 |

Note: *, *p*＜0.05; **, *p*＜0.01

**Table S5** Contribution of soil properties to variation of leaf traits of *P. harmala*.

| Soil variables | Explains (%) | Contribution (%) | Pseudo-F | *p* |
| --- | --- | --- | --- | --- |
| C | 8.1 | 18.2 | 1.7 | 0.164 |
| VFS | 8.4 | 18.7 | 1.6 | 0.164 |
| MS | 6.0 | 13.4 | 1.2 | 0.290 |
| CS | 3.9 | 8.8 | 0.8 | 0.474 |
| pH | 3.6 | 8.1 | 0.7 | 0.530 |
| WC | 3.4 | 7.7 | 0.7 | 0.548 |
| SC | 3.0 | 6.7 | 0.5 | 0.680 |
| EC | 2.1 | 4.6 | 0.4 | 0.760 |
| BD | 2.2 | 5.0 | 0.4 | 0.762 |
| FS | 2.3 | 5.1 | 0.4 | 0.770 |
| N | 1.6 | 3.6 | 0.2 | 0.888 |

## Supplementary Figure


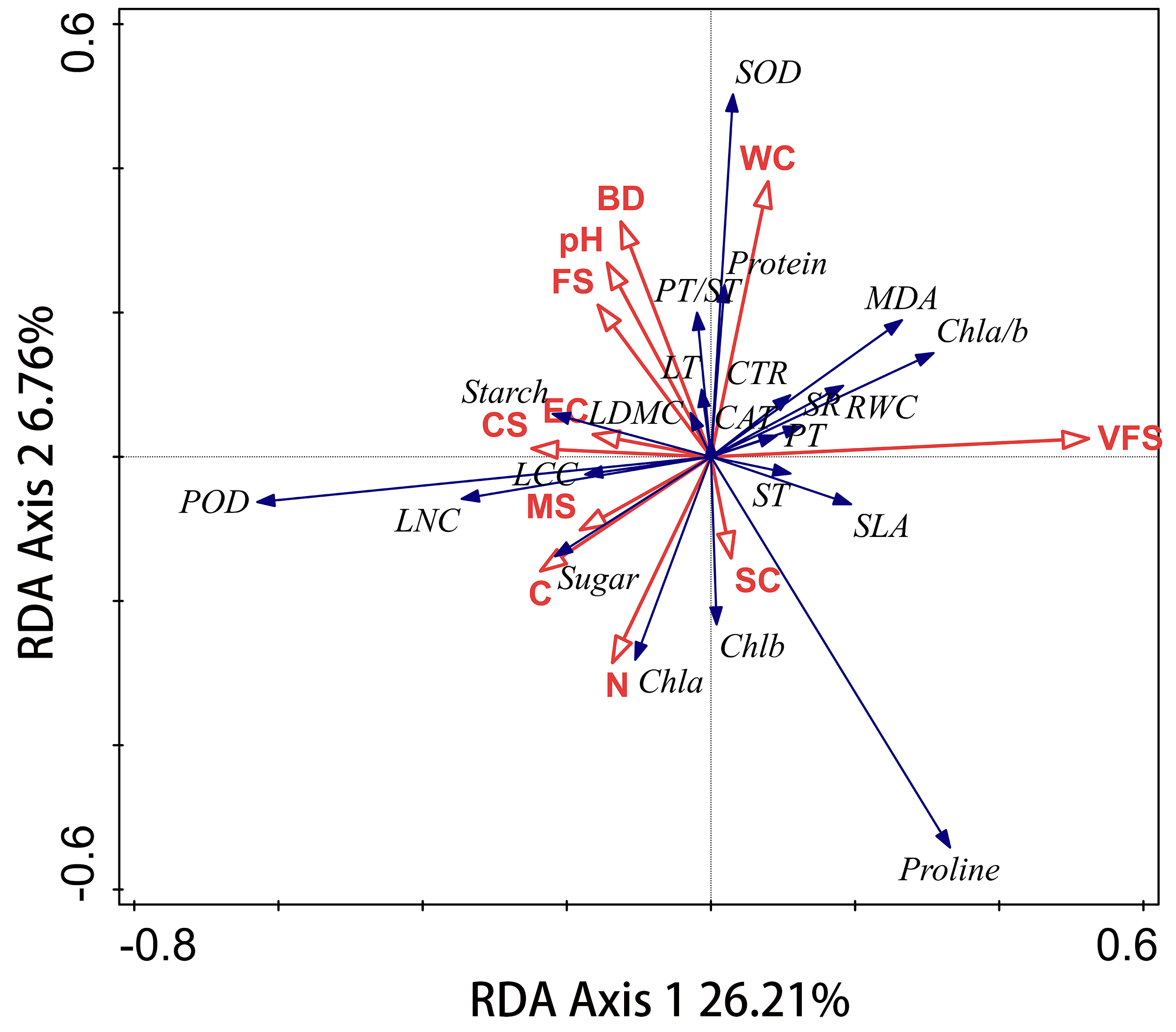


**Figure S1**. Redundancy analysis indentifying the effects of soil properties on leaf traits of *P. harmala*.
